# Dietary Vitamin D Intake, Pain Incidence, and Pain Changes in Older Adults: The Seniors-ENRICA-1 Cohort

**DOI:** 10.3390/nu14183776

**Published:** 2022-09-13

**Authors:** Adrián Carballo-Casla, Sonia de Paz-Cantos, Rosario Ortolá, Esther García-Esquinas, Mercedes Sotos-Prieto, José R. Banegas, Fernando Rodríguez-Artalejo

**Affiliations:** 1 Department of Preventive Medicine and Public Health, Universidad Autónoma de Madrid, Calle del Arzobispo Morcillo 4, 28029 Madrid, Spain; 2 CIBER of Epidemiology and Public Health (CIBERESP), Avenida de Monforte de Lemos 3-5, 28029 Madrid, Spain; 3 Department of Environmental Health, Harvard T.H. Chan School of Public Health, 665 Huntington Avenue, Boston, MA 02115, USA; 4 IMDEA Food Institute, CEI UAM+CSIC, Carretera de Canto Blanco 8, 28049 Madrid, Spain

**Keywords:** sunlight, micronutrient, calcium, vitamin D supplements, serum vitamin D, low back, pain treatment, pain intensity, longitudinal, elderly

## Abstract

Background: Vitamin D plays a role in bone health, pain signaling, and inflammation. We examined the largely unknown relation of dietary vitamin D intake with pain incidence and pain changes over time in older adults. Methods: Data were taken from the Seniors-ENRICA-1 cohort, which included 950 individuals aged ≥60 years. Habitual vitamin D intake was assessed in 2012 with a validated diet history, and pain both in 2012 and 2017 with a scale ranging from 0 (no pain) to 6 (highest pain), according to its severity, frequency, and number of locations. Analyses on pain incidence and pain changes were performed in the 524 participants free of pain at baseline and the overall sample, respectively. Results: Higher dietary vitamin D intake was associated with lower 5-year pain incidence; the multivariable-adjusted odds ratio (95% confidence interval) was 0.88 (0.79,0.99) for every 1-µg/day increase in vitamin D intake, and 0.49 (0.28,0.88) for the highest (>3.52 µg/day) vs. lowest (<1.85 µg/day) tertile. Dietary vitamin D intake (highest vs. lowest tertile) was also associated with 5-year favorable pain changes: the multivariable-adjusted odds ratio of pain worsening vs. no change/pain improvement was 0.55 (0.36,0.86), and the β coefficient for changes in the pain scale was −0.56 (−1.03,−0.09). Similar results were found for pain severity, frequency, and number of pain locations. Conclusions: In an older adult population, where compliance with vitamin D intake recommendations was very low, a slightly increased dietary intake was associated with lower pain incidence and favorable pain changes over 5 years.

## 1. Introduction

The number of people who suffer from pain is increasing steadily in the world. Namely, the number of years lived with disability caused by low back pain rose by 54% between 1990 and 2015 [1]. The prevalence of chronic pain is also high, ranging from 10% to 65% of adults in the United States, and affecting around 1.5 billion people worldwide -more than diabetes, cancer, and heart disease combined [2,3,4]. The global burden of pain is enormous, as low back pain in the US alone is estimated to cost between $560 billion and $635 billion per year in health care use, disability, and lost productivity [1,5]. These figures highlight the need for better pain prevention and management strategies, especially in older adults, as (1) most burden of pain is concentrated in the older population, (2) chronic pain affects up to 60% of people >65 years, and (3) the former is expected to increase parallel to the word population aging [6,7,8].

Unfortunately, few effective pain prevention strategies exist, being mostly physical activity and avoidance of sedentary behavior [3,9]. Moreover, despite some advancements, pain management continues to be suboptimal [1], and there are concerns about the side effects of pain medications, such as opioids or non-steroidal anti-inflammatory drugs [9]. Pharmacological management of pain in older adults may be further compromised by polypharmacy, excess toxicity, and risks on cognition and several organ systems [10,11].

A growing body of evidence supports foods and nutrients as a potential means to prevent and reduce pain [12,13]. Among the most promising is vitamin D, for it plays a fundamental role on bone health [14], might modulate pain signaling pathways [15], has shown anti-inflammatory effects [16], and acts on sleep regulatory mechanisms, whose malfunction may lead to hyperalgesia [17]. Low serum vitamin D concentrations are frequent among older adults [18] and have also been associated with conditions such as widespread pain, muscle pain, arthritis, and low-back pain [19,20].

Although sun exposure is the main source of bodily vitamin D, this synthesis pathway is highly dependent on season, latitude, and epidermal pigmentation [21,22]. Moreover, clothing habits of older adults may not allow the sun to come into direct contact with the skin, and both bodily vitamin D synthesis and vitamin D action are impaired in old age [21,23], so sufficient intake remains essential, especially in this population group. Nevertheless, vitamin D intake recommendations (≥15 µg/day) are rarely attained in older adults [24], as foods rich in vitamin D are scarce, and most studies examining vitamin D influences on pain-related outcomes have been conducted as rather small-sized, short-term interventions relying on vitamin D supplements [25,26]. Although such trials have generally increased serum vitamin D levels and lowered pain intensity [23,25,27], the use of very high vitamin D doses might not grant additional clinical benefits [15,27]. Moreover, increased sample size and especially longer-term follow-up would be desirable to determine further clinical effects on pain, and the role of vitamin D as a pain prevention strategy remains largely unexamined [9,15,25,27].

### Objectives

Accordingly, the main objectives of this investigation were to study the association of habitual dietary vitamin D intake with (1) pain incidence and (2) pain changes over a 5-year follow-up in a cohort of community-dwelling older adults. We further examined the association between dietary vitamin D intake and the three main components of pain: severity, frequency, and number of locations [6,28].

## 2. Methods

### 2.1. Study Design and Participants

Our data came from the Seniors-ENRICA-1 study, a cohort of community-dwelling adults ≥60 years in Spain (ClinicalTrials.gov Identifier: NCT01133093) [29,30]. Subjects were recruited between March 2008 and September 2010 by stratified cluster sampling and followed-up at February-to-November 2012 and January-to-July 2017. Since information on pain was not collected in 2008–2010, for this study, 2012 was considered the baseline assessment.

Data were collected in 2012 and 2017 in two stages. First, data on pain, sociodemographic variables, lifestyle, and morbidity were obtained by telephone interviews. Second, a detailed, validated dietary history was obtained and a physical examination was performed during home visits by trained personnel [30]. Vitamin D intake was derived from the diet history conducted in 2012 [31], whereas pain was assessed in 2012 and 2017 using a scale derived from the large Survey on Chronic Pain in Europe [6,28].

The study was approved by the Clinical Research Ethics Committee of *La Paz University Hospital* in Madrid (protocol code HULP: PI-1793) and all participants gave informed written consent.

### 2.2. Study Variables

#### 2.2.1. Vitamin D Intake

Food consumption was obtained through a face-to-face validated computerized dietary history [31]. Participants could report up to 880 food and beverages habitually consumed in the previous year. Color photographs were used to quantify portion sizes. To convert foods to energy and nutrients, including vitamin D, the computerized dietary history used data from six food composition tables from Spain and five tables from other countries [31]. A previous validation study comparing the results of this diet history against seven 24-h recalls over one year showed a mean correlation coefficient of 0.76 for energy and 0.30 for vitamin D [31].

#### 2.2.2. Pain

To assess pain, we used a 10-question-scale developed from the Survey on Chronic Pain in Europe, which comprised three components [6,28]: severity, frequency, and number of pain locations. (1) Pain severity was estimated according to its influence on the activities of daily living. Those who had moderately, much, or completely troublesome pain were deemed to suffer from moderate–high pain, whereas those with little or no trouble because of pain were classified as having light pain. (2) Regarding frequency, participants were considered to have persistent pain (≥2 times/week), sporadic pain (≤1 time/week), or to be pain-free (no pain in the previous 6 months). (3) Six pain locations were considered: head and neck, back, bones and joints, legs, arms, and others (reported by 34.9%, 50.1%, 61.6%, 64.2%, 42.9%, and 24.6% of the subjects with pain in 2012, respectively). Subjects were then classified according to the number of pain sites (0, 1–2, and ≥3).

Sporadic vs. persistent pain, light vs. moderate-high pain, and 1–2 vs. ≥3 pain sites were assigned a score of 1 and 2 points, respectively. The pain scale was then built as the sum of said three components and ranged from 0 (no pain in the previous six months) to 6 (highest pain). We defined incident pain as the presence of pain in 2017 among the subjects who had scored 0 points on the pain scale in 2012. We also calculated changes in the pain scale from 2012 to 2017, so that negative values indicated pain improvement and vice-versa. Subjects who maintained or decreased their pain scale score between 2012 and 2017 (no change/pain improvement) and those who increased it (pain worsening) reported a mean change in the pain scale of −1.33 and 3.66 points, respectively.

#### 2.2.3. Potential Confounders

Data on several potential confounders were collected, specifically sex, age, educational level (≤primary, secondary, or university), tobacco smoking (never, former, or current), and alcohol consumption [never, former, moderate (≤10 g/day in women and ≤20 g/day in men), or heavy drinking]. Recreational physical activity [Metabolic Equivalents of Task-hour/week (MET-hour/week)] was assessed with the EPIC-cohort questionnaire validated in Spain [32]. Sedentary behavior (hours/week) was approximated to time spent watching television via the Nurses’ Health Study questionnaire validated in Spain [33,34]. Weight (kg) and height (m) were measured under standardized conditions, and the body mass index (BMI) was calculated as the former divided by the latter squared [35]. Concerning morbidity, subjects were deemed to have diabetes if they were treated with antidiabetic drugs, reported that their doctor had given them a diabetes diagnosis, or had blood glucose levels ≥ 126 mg/dL in the 2008–2010 wave (note that we did not collect blood samples in 2012). Participants also reported the following physician-diagnosed diseases: cardiovascular disease (coronary heart disease, stroke, or heart failure), chronic obstructive pulmonary disease, musculoskeletal disease (osteoarthritis, arthritis, or hip fracture), cancer, and depression requiring medical treatment [30]. Potential dietary confounders [energy (kcal/day), fiber (g/day), carbohydrate (g/day), unsaturated fatty acids (g/day), and sodium intake (mg/day)] were obtained from the dietary history [31].

### 2.3. Statistical Methods

#### 2.3.1. Study Size

From the 2519 subjects followed in 2012, we excluded 196 individuals who had died and 1185 who were lost to follow-up in 2017. From the remaining 1138 subjects, we further excluded 188 with incomplete data (96 subjects had no information on vitamin D, 95 on pain variables, and 98 on potential confounders), leaving 950 individuals in the main analytical sample. For the analyses regarding pain incidence, we further excluded 426 subjects who already had pain at the 2012 wave. Therefore, this secondary analytical sample comprised 524 subjects (Supplementary Figure S1).

Compared with the participants who were included in the main analytic sample (n = 950), those excluded (n = 1569) were older (73.4 vs. 70.7 years) and less educated (58.3% vs. 48.3% had primary studies), and showed some differences in other variables, namely former drinking (18.7% vs. 15.3%), physical activity (21.0 vs. 22.6 MET-hour/week), sedentary behavior (2.88 vs. 2.68 television hours/day), number of chronic diseases (1.33 vs. 1.15), and energy (1978 vs. 2041 kcal/day), fiber (24.5 vs. 25.3 g/day), unsaturated fat (48.9 vs. 51.6 g/day), and sodium (2605 vs. 2697 mg/day) intake.

#### 2.3.2. Statistical Methods

Differences in characteristics of study participants across the categories of dietary vitamin D intake were evaluated with Pearson’s chi-squared tests for categorical variables and analysis of variance (ANOVA) for continuous variables.

The associations of dietary vitamin D intake with 5-year (1) incident pain and (2) changes in the pain scale categories (pain worsening vs. no change/pain improvement) were summarized with odds ratios (OR) and their 95% confidence intervals (95% CI), obtained from logistic regression. Those between dietary vitamin D intake and 5-year changes in the pain scale scores were summarized with beta coefficients (β) and their 95% CI, obtained from linear regression. Similar analyses were performed for the associations with changes in the components of the pain scale.

To control for potential confounding, three incrementally adjusted models were used. The first, adjusted for sex, age, and educational level. The second, additionally adjusted for smoking status, alcohol consumption, leisure-time physical activity, sedentary behavior, BMI, energy intake, and number of chronic diseases. The third, additionally adjusted for fiber, carbohydrate, unsaturated fat, and sodium intake.

Dietary vitamin D intake was analyzed as: (1) a continuous variable (per 1-µg/day increment), (2) tertiles, using the lowest as reference (Tertile 1, 0.01–1.85 µg/day; Tertile 2, 1.85–3.51 µg/day; Tertile 3, 3.52–24.5 µg/day), and (3) a restricted-cubic spline (knots located at the 10th, 50th, and 90th percentiles [36]).

#### 2.3.3. Interactions and Sensitivity Analyses

We used Wald tests to examine whether the associations of dietary vitamin D intake with pain were modified by other variables (i.e., we fitted models with multiplicative interaction terms and tested whether the interaction coefficients were equal to zero). Several effect modifiers were considered: (1) sex, age, tobacco smoking, recreational physical activity, and BMI, as pain prevalence has been found to vary across sexes [6,7,8], while the latter four variables are important determinants of pain incidence and pain evolution [1,3,37]; and (2) the average provincial sunshine hours and time spent sunbathing, as there is a known association between sun exposure and serum vitamin D levels [22]. For this purpose, we used 2011–2015 data compiled by the Spanish Meteorological Agency [38], and self-reported time spent sunbathing (either sitting or lying, both in winter and summer time) [34]. Since there were no statistically significant interactions, results are presented for the total sample.

We also conducted four sensitivity analyses. Here, we adjusted the models for several variables not included in the main analyses, to check for residual confounding or mediation, where appropriate. The candidate variables were: (1) calcium intake, as vitamin D increases intestinal calcium absorption and both nutrients play a joint role on bone health [14,23]; (2) sleep quantity and quality, because vitamin D acts in sleep regulatory mechanisms, whose malfunction may lead to hyperalgesia [17]; (3) baseline use of pain medications, given that some mechanisms of action of painkillers may overlap with those of vitamin D (i.e., modulation of pain signaling pathways [15] and anti-inflammatory effects [16]); and (4) vitamin D supplement use, as vitamin D pills’ dosing likely outweighs foods’ [24,39]. For this same reason, we ran a final analysis excluding those subjects taking said supplements. Calcium intake was taken from the dietary history [31]; nocturnal sleep was self-reported, and sleep quality was based on the Epworth Sleepiness Scale [40]; pain medications and vitamin D supplements were self-reported and checked by the study staff against drug packages at home, respectively [30].

Statistical significance was set at two-tailed *p* < 0.05. Analyses were conducted using Stata/SE, version 16 (StataCorp, College Station, TX, USA).

## 3. Results

### 3.1. Descriptive Data

The mean (standard deviation) dietary vitamin D intake was 3.24 (2.47) µg/day, and the food groups contributing the most to it were fish, especially oily fish, eggs, meat, cereals, and dairy (Supplementary Table S1). Table 1 shows the sociodemographic, lifestyle, and dietary characteristics of the study participants. In the main analytical sample, comprising 950 subjects with and without pain, those with higher dietary vitamin D intake were younger, more often men and former smokers, engaged in more physical activity, and had higher energy, fiber, carbohydrate, unsaturated fat, and sodium intake. Within the 524 pain-free subjects at the 2012 wave, differences were similar, except that the subjects with higher vitamin D intake were less frequently never drinkers and there were no disparities in tobacco smoking.

### 3.2. Main Results

**Figure 1:**
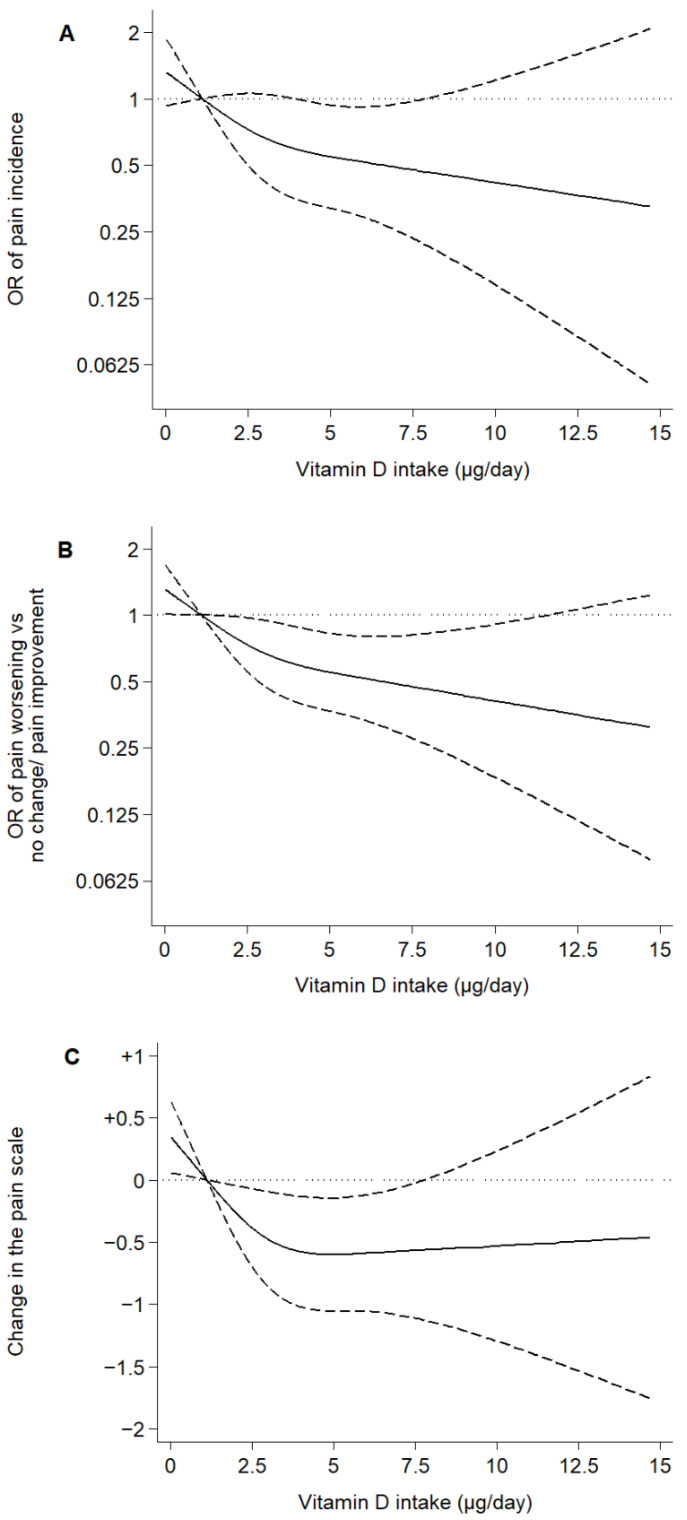
Associations of dietary vitamin D intake with pain incidence and changes in the pain scale over 5 years in older adults. Notes: Plotted values are odds ratios (95% confidence intervals) from a logistic regression model as Model 3 in Table 2 (**A**,**B**), or β coefficients (95% confidence interval) from a linear regression model as Model 6 in Table 2 (**C**) [adjusted for sex, age, educational level (primary or less, secondary, or university), smoking status (never, former, or current), alcohol consumption (never, former, moderate, heavy), leisure-time physical activity (MET-hours/week), sedentary behavior (television hours/day), body mass index (kg/m^2^), energy intake (kcal/day), number of chronic diseases (diabetes, cardiovascular disease, chronic lung disease, musculoskeletal disease, cancer, and depression), fiber, carbohydrate, unsaturated fat, and sodium intake]. Restricted cubic spline knots: 0.93, 2.67, and 5.77 µg/day. Reference: 0.96 µg/day.

After a mean follow-up time of 4.92 years, 125 pain-incident and 184 pain-worsening cases were ascertained. Higher dietary vitamin D intake was associated with a decrease in pain incidence and favorable pain changes. Specifically, the model 3 OR (95% CI) for pain incidence was 0.88 (0.79,0.99) for every 1-µg/day increase in dietary vitamin D intake and 0.49 (0.28,0.88) for the highest vs. lowest tertile of intake. The model 3 OR (95% CI) of pain worsening vs. no change/pain improvement and the β for the change in the pain scale scores were 0.55 (0.36,0.86) and −0.56 (−1.03,−0.09) for the highest vs. lowest tertile of dietary vitamin D intake, respectively (Table 2). A clear dose–response relationship was observed when plotting dietary vitamin D intake as a restricted cubic spline (Figure 1); no significant departures from linearity were observed (*p* for nonlinear trend were 0.37, 0.27, and 0.06 for pain incidence, pain worsening vs. no change/pain improvement, and changes in the pain scale, respectively).

**Figure 2:**
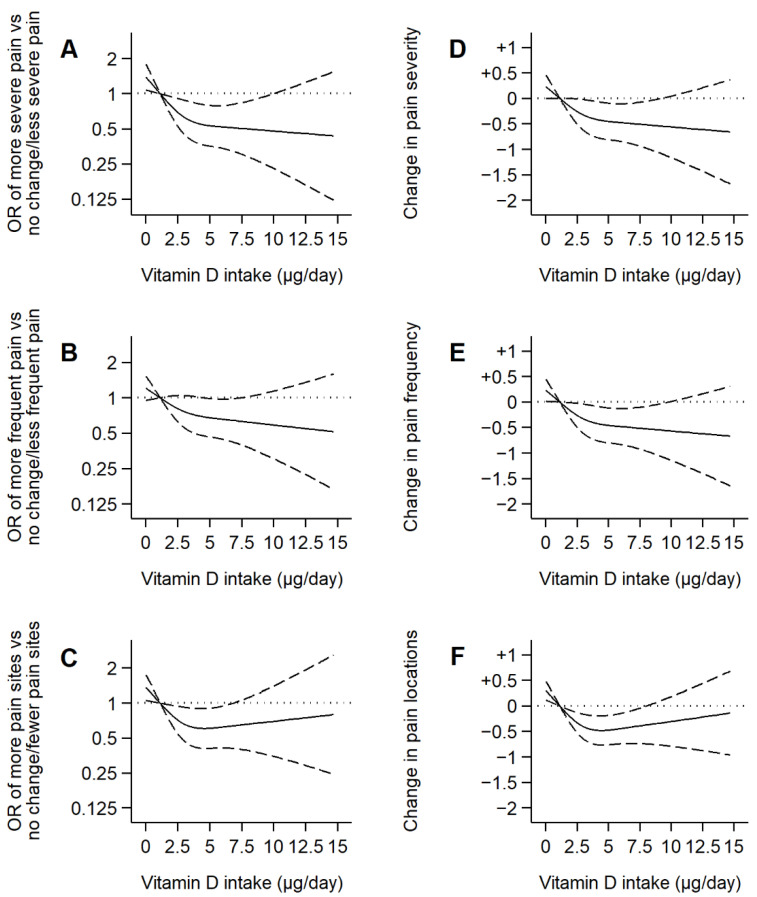
Associations of dietary vitamin D intake with changes in the components of the pain scale over 5 years in older adults. Notes: Plotted values are odds ratios (95% confidence intervals) from a logistic regression model as Model 3 in Table 3 (**A**,**B**,**C**), or β coefficients (95% confidence interval) from a linear regression model as Model 6 in Table 3 (**D**,**E**,**F**) [adjusted for sex, age, educational level (primary or less, secondary, or university), smoking status (never, former, or current), alcohol consumption (never, former, moderate, heavy), leisure-time physical activity (MET-hours/week), sedentary behavior (television hours/day), body mass index (kg/m^2^), energy intake (kcal/day), number of chronic diseases (diabetes, cardiovascular disease, chronic lung disease, musculoskeletal disease, cancer, and depression), fiber, carbohydrate, unsaturated fat, and sodium intake]. Restricted cubic spline knots: 0.93, 2.67, and 5.77 µg/day. Reference: 0.96 µg/day.

Similar results were found when examining the pain scale components. A 1-µg/day increase in dietary vitamin D intake was associated with a beneficial 5-year change in pain severity [β coefficient (95% CI): −0.06 (−0.12,−0.00)], pain frequency [−0.06 (−0.12,−0.00)], and number of pain locations [−0.05 (−0.10,−0.00)] (Table 3, Figure 2).

### 3.3. Interactions and Sensitivity Analyses

We found no evidence that either sex, age, tobacco smoking, recreational physical activity, BMI, the average provincial sunshine hours, or time spent sunbathing significantly modified the associations of dietary vitamin D intake with pain incidence and pain changes (Supplementary Figure S2). These associations were also consistent when adjusting the analyses for: (1) calcium intake; (2) sleep quantity and quality; (3) the baseline use of pain medications; and (4) vitamin D supplement use, as well as when excluding the subjects taking said supplements (Supplementary Table S2).

## 4. Discussion

In this cohort of Spanish older adults, higher dietary vitamin D intake was associated with lower pain incidence and favorable pain changes over 5 years. The three components of the pain scale seemed to contribute to these findings, including pain severity -that pain affecting the activities of daily living. Results were consistent in several sensitivity analyses.

### 4.1. Interpretation

#### 4.1.1. Relevant Findings from Other Published Studies

The pooled results of randomized controlled trials regarding vitamin D supplementation on pain show: (1) a significantly greater mean decrease in the visual analog pain scale (VAS) -though such differences were only observed in hospitalized patients with pain-related medical conditions; (2) no differences in the final VAS; and (3) a non-significant effect for pain improvement vs. no change/pain worsening. Similar results were found when analyzing studies on widespread and localized pain separately [25]. A subsequent meta-analysis confirmed these results on widespread pain patients, though the increase in serum vitamin D concentrations in the treatment groups was not significantly related with a lowering in the VAS [41]. Conversely, a meta-analysis on low back pain indicated that vitamin D supplementation had neither an effect on pain intensity, nor on pain improvement vs. no change/pain worsening [26].

Regarding meta-analyses of observational evidence, mean serum vitamin D concentration was lower and the odds of vitamin D deficiency were higher in patients with arthritis, muscle pain, widespread pain, and low back pain (especially severe vs. mild low back pain) [19,20]. Nevertheless, results from vitamin D supplement trials and observational studies on serum vitamin D and pain may differ from those on habitual dietary vitamin D intake. On one hand, the relation between vitamin D intake and its serum levels might be nonlinear, and the use of very high vitamin D doses might not therefore grant additional clinical benefits [27]. On the other hand, serum vitamin D levels are not exclusively dependent on food consumption and supplement use, as the endogenous production, occurring in the skin and dependent on sun exposure, contributes to most of the bodily vitamin D levels [42]. Despite these potential differences, nearly all studies on habitual dietary vitamin D intake have focused on pain-related conditions rather than pain itself. Namely, one meta-analysis showed a beneficial association between total vitamin D intake and rheumatoid arthritis incidence [43], while the former was also related to a decreased risk of incident endometriosis in a US cohort [44].

#### 4.1.2. Possible Mechanisms and Explanations

The pathophysiological mechanisms linking vitamin D and pain may be threefold. First, there is a potential interplay between vitamin D and pain signaling pathways [15]. Specifically, vitamin D and vitamin D receptor might play a role in lowering pain-sensing through modulating key pain-genes, which are involved in the development of Schwann cells and nociceptor neurons. This role is biologically plausible, as both vitamin D and/or vitamin D receptor genes are expressed in tissues such as skin, dorsal root ganglion neurons, spinal cord, and brain. Additionally, vitamin D may change the gut microbiota profile and potentially modulate visceral pain—note that the latter might stem from microbes and metabolites secreted or degraded by the gut microbiome [15].

Second, vitamin D has shown anti-inflammatory effects [15,16]. On one hand, vitamin D shifts the T-cell response away from the pro-inflammatory T-helper type cells. On the other hand, it inhibits the synthesis, release, or transactivation of prostaglandin E2, several inflammatory cytokines, toll-like receptors, and the epidermal growth factor receptor [15,16]. These mechanisms may explain why the beneficial associations of dietary vitamin D intake with pain incidence and pain changes were somewhat more marked in current vs. former/never smokers (Supplementary Figure S2), as tobacco smoke has been shown to augment the production of pro-inflammatory cytokines and reduce that of anti-inflammatory cytokines [45].

Third, vitamin D possibly modulates sleep regulatory mechanisms, whose malfunction may lead to hyperalgesia [17]. On one hand, the neuronal expression of vitamin D and/or vitamin D receptor genes takes place in brain regions that regulate the sleep–wake cycle. On the other hand, the association between low serum vitamin D concentrations and sleep disorders is rather well-established, and the latter may lead to a reduction in serotonin release, contributing to nociceptive pain stimulation. Finally, changes in sleep patterns and certain sleep disorders may lead to pain sensitivity and trigger painful conditions, while restorative sleep is a predictor of pain resolution [17]. Nevertheless, we have been unable to hint a modulation of the association between dietary vitamin D intake and pain by sleep regulatory mechanisms, as adjusting the analyses for sleep quantity and quality did not materially change the estimates (Supplementary Table S2).

### 4.2. Limitations

First, the correlation between the dietary vitamin D intake estimated via our diet history and seven 24-h recalls over one year was only moderate (r = 0.30) [31]. This is consistent with the fact that a few foods groups account for most of the vitamin D intake (Supplementary Table S1), and these may have been underreported in the dietary history because of recall error. Nevertheless, there was little gross vitamin D misclassification between the dietary history and said 24-h recalls, as the percentage of subjects simultaneously classified in the lowest quintile by the latter method and the highest quintile by the former was 5.0%, while it was 5.3% for the opposite situation [31]. It is also encouraging to see how modelling dietary vitamin D intake as a continuous (per 1-µg/day increment) and categorical variable (tertiles) rendered consistent results (Table 2).

Second, our pain questionnaire and scale have not been validated, and they do not make distinctions between etiologies and types of pain (e.g., neuropathic, nociceptive), whose biological mechanisms may differ. Nevertheless, the items were similar to those used in the Survey on Chronic Pain in Europe [6], and the changes in the pain scale were consistent with those of other widely used instruments [46]. For example, participants who maintained or decreased their pain scale score from 2012 to 2017 and those who increased it reported an average pain intensity change of −1.56 and 4.15 points [in a Numeric Rating Scale from 1 (no pain) through 10 (a pain I cannot even imagine bearing)], respectively. Additionally, the self-reported nature of most covariates may not allow residual confounding to be ruled out, even after adjusting the regression models for many sociodemographic, lifestyle, morbidity, and diet-related variables. For instance, vitamin D supplement uptake (by 4.32% of the study subjects) may have been underreported, and we lacked the dosing data eventually needed to combine vitamin D pills’ intake with foods’. Then again, it is reassuring to see that results for minimally-adjusted vs. fully-adjusted models, those adjusted for vitamin D supplement use, and the analyses excluding the subjects taking said supplements were not much different (Supplementary Table S2).

Third, the somewhat high loss to follow-up rate (Supplementary Figure S1) led to: (1) a selection of younger, more educated, and globally healthier subjects, which may have biased the study results in any way; and (2) some imprecision due to the limited sample size, especially in the analyses of incident pain (n = 524).

### 4.3. Generalizability

To what extent do our estimates apply to other populations and settings? First, Spain is a Southern European country where the Mediterranean climate is king. Summers are warm/hot and dry, and more than half of our participants lived in regions with >7 daily sunshine hours (range: 4.1–9.1) [38,47]. Since there is a known association between sun exposure and serum vitamin D levels [22], our results may differ from those of cooler and cloudier countries. Nevertheless, (1) said results held countrywide, regardless of the average provincial sunshine hours and sunbathing habits (Supplementary Figure S2); and (2) the older adults living in Southern European countries have low serum vitamin D levels more often than those living in Northern Europe [21]. This may be explained twofold: first, it is common for older adults in the south to spend little time under the sun -note that only 5% of our subjects reported sunbathing in winter and 21% in summertime-, and clothing habits may not allow the sun to come into direct contact with the skin; second, dairy products, edible fats, and oils are compulsorily enriched with vitamin D in many Northern European countries, but not in their Southern counterparts [21]. Since we observed a strong dose–response relationship for the association between vitamin D intake and pain (Figure 1), we hypothesize that our results may also be relevant for those countries with higher dietary vitamin D intake but, at the same time, said results may not be generalized to vitamin D supplement users, who often exceed the recommended 15 µg/day intake. Note that only 0.42% of our study subjects were above this threshold- [24,39].

Second, our study population was over 60 years old, and both pain frequency and vitamin D deficiency steadily increase with age [7,48]. On one hand, we were not able to examine whether the relationship between dietary vitamin D intake and pain was modified and/or mediated via the baseline serum vitamin D levels, as we lacked the corresponding data. On the other hand, we found no evidence that age significantly modified the study associations, but their strength seemed to increase somewhat with increasing age (Supplementary Figure S2). Lastly, the Seniors-ENRICA-1 population was almost entirely white (99.2%), thus warranting the need for caution when applying our results to multiethnic/multiracial populations, as people who are dark-skinned are more likely to suffer from hypovitaminosis D than Caucasians [22].

### 4.4. Conclusions

In this cohort of Spanish older adults, where compliance with vitamin D intake recommendations was very low, a slightly increased dietary intake was associated with lower pain incidence and favorable pain changes over 5 years. These associations were consistent in main and sensitivity analyses and highlight the potential role of dietary vitamin D intake as an adjunctive pain prevention and management strategy.

Given that diet is often measured with some error, can change over time, and its effects on health could be cumulative, larger observational studies with repeated assessment of vitamin D intake should replicate these findings. Future trials should also assess the effectiveness of pain prevention and management interventions targeting the main dietary sources of vitamin D intake (e.g., fish and eggs).

## Figures and Tables

**Table 1:** Baseline characteristics of 524 older adults without pain and 950 older adults with and without pain, by categories of dietary vitamin D intake.

	Vitamin D Intake (Subjects without Pain) ^a^			Vitamin D Intake (Subjects with and without Pain) ^a^		
	Tertile 1 (Lowest)	Tertile 2	Tertile 3 (Highest)	Tertile 1 (Lowest)	Tertile 2	Tertile 3 (Highest)
**n**	156	188	180	278	345	327
Vitamin D (µg/day)	1.16 (0.46)	2.56 (0.48)	5.60 (2.60)	1.15 (0.43)	2.59 (0.48)	5.69 (2.67)
Sex-Men, n (%)	69 (44.2)	110 (58.5)	119 (66.1) *	108 (38.9)	164 (47.5)	188 (57.5) *
Age (years)	71.9 (5.81)	70.8 (5.37)	70.1 (5.13) *	71.3 (5.64)	70.7 (5.22)	70.1 (4.98) *
Educational level, n (%)						
Primary or less	70 (44.9)	85 (45.2)	68 (37.8)	147 (52.9)	165 (47.8)	147 (45.0)
Secondary	47 (30.1)	44 (23.4)	58 (32.2)	71 (25.5)	89 (25.8)	97 (29.7)
University	39 (25.0)	59 (31.4)	54 (30.0)	60 (21.6)	91 (26.4)	83 (25.4)
Tobacco smoking, n (%)						
Never	96 (61.5)	107 (56.9)	86 (47.8)	182 (65.5)	206 (59.7)	171 (52.3) *
Former	47 (30.1)	69 (36.7)	77 (42.8)	75 (27.0)	109 (31.6)	130 (39.8)
Current	13 (8.33)	12 (6.38)	17 (9.44)	21 (7.55)	30 (8.70)	26 (7.95)
Alcohol consumption, n (%)						
Never	33 (21.2)	35 (18.6)	16 (8.9) *	61 (21.9)	71 (20.6)	43 (13.2)
Former	75 (48.1)	79 (42.0)	81 (45.0)	115 (41.4)	145 (42.0)	138 (42.2)
Moderate ^b^	29(18.6)	53 (28.2)	55 (30.6)	60 (21.6)	81 (23.5)	91 (27.8)
Heavy	19 (12.2)	21 (11.2)	28 (15.6)	42 (15.1)	48 (13.9)	55 (16.8)
Physical activity, MET-hours/week	20.6 (14.4)	24.2(14.7)	26.0 (16.2) *	20.4 (14.3)	22.3 (14.7)	24.9 (15.8) *
Sedentary behavior, TV hours/day	2.59 (1.24)	2.44 (1.40)	2.48 (1.36)	2.80 (1.54)	2.59 (1.36)	2.66 (1.49)
Energy intake, kcal/day	1907 (400)	2000 (404)	2173 (510) *	1898 (381)	2018 (432)	2188 (508) *
Body mass index, kg/m^2^	27.9 (4.28)	27.9 (3.91)	27.8 (3.85)	28.2 (4.46)	28.4 (4.30)	28.3 (4.28)
Number of chronic diseases ^c^	0.99 (0.49)	0.98 (0.88)	0.81 (0.87)	1.24 (0.99)	1.17 (0.93)	1.06 (0.98)
Fiber intake, g/day	23.5 (6.54)	23.9 (7.14)	26.4 (8.82) *	23.9 (7.00)	24.4 (7.44)	27.6 (9.80) *
Carbohydrate intake, g/day	202 (43.8)	203 (44.3)	216 (57.5) *	203 (42.8)	209 (48.4)	220 (59.8) *
Unsaturated fat intake, g/day	47.7 (14.3)	50.7 (15.1)	57.0(17.3) *	46.6 (14.1)	50.6 (15.8)	56.9 (16.8) *
Sodium intake, mg/day ^d^	2437 (810)	2650 (915)	2817 (1062) *	2438 (792)	2693 (1004)	2921 (1064) *

Values are numbers (%) or means (standard deviations). * *p*-value < 0.05 for differences in means (ANOVA) or proportions (Pearson’s chi-squared) across tertiles of vitamin D intake. ^a^ Vitamin D intake categories: Tertile 1, 0.01 to 1.85 µg/day; Tertile 2, 1.85 to 3.51 µg/day; Tertile 3, 3.52 to 24.46 µg/day. ^b^ Moderate drinking: ≤10 g/day in women and ≤20 g/day in men. ^c^ Diabetes, cardiovascular disease, chronic lung disease, musculoskeletal disease, cancer, and depression. ^d^ Salt added during cooking and at the table not included.

**Table 2:** Associations of dietary vitamin D intake with pain incidence and changes in the pain scale over 5 years in older adults.

	Vitamin D Intake ^a^			
	Tertile 1 (Lowest)	Tertile 2	Tertile 3 (Highest)	Per 1-µg/day Increment
**Pain incidence**				
Cases/n	48/156	46/188	31/180	125/524
Model 1: OR (95% CI) ^b^	Ref.	0.80 (0.49,1.30)	0.54 (0.32,0.92) *	0.89 (0.81,0.99) *
Model 2: OR (95% CI) ^c^	Ref.	0.78 (0.47,1.30)	0.52 (0.29,0.92) *	0.89 (0.80,1.00) *
Model 3: OR (95% CI) ^d^	Ref.	0.78 (0.47,1.32)	0.49 (0.28,0.88) *	0.88 (0.79,0.99) *
**Changes in the pain scale**				
Pain worsening vs. no change/pain improvement				
Cases/n	69/278	66/345	49/327	184/950
Model 1: OR (95% CI) ^b^	Ref.	0.75 (0.51,1.10)	0.59 (0.39,0.89) *	0.89 (0.82,0.97) **
Model 2: OR (95% CI) ^c^	Ref.	0.74 (0.50,1.09)	0.57 (0.37,0.88) *	0.88 (0.81,0.96) **
Model 3: OR (95% CI) ^d^	Ref.	0.73 (0.49,1.08)	0.55 (0.36,0.86) **	0.88 (0.81,0.96) **
Change in the pain scale				
n	278	345	327	950
Model 4: β (95% CI) ^e^	Ref.	−0.56 (−1.00,−0.11) *	−0.57 (−1.03,−0.12) *	−0.07 (−0.15,−0.00) *
Model 5: β (95% CI) ^f^	Ref.	−0.54 (−0.99,−0.10) *	−0.53 (−1.00,−0.06) *	−0.07 (−0.14,0.01)
Model 6: β (95% CI) ^g^	Ref.	−0.56 (−1.01,−0.12) *	−0.56 (−1.03,−0.09) *	−0.07 (−0.15,0.00)

* *p* < 0.05. ** *p* < 0.01. OR: odds ratio. CI: confidence interval. ^a^ Vitamin D intake categories: Tertile 1, 0.01 to 1.85 µg/day; Tertile 2, 1.85 to 3.51 µg/day; Tertile 3, 3.52 to 24.46 µg/day. ^b^ Model 1: Logistic regression model adjusted for sex, age, and educational level (primary or less, secondary, or university). ^c^ Model 2: As Model 1 and additionally adjusted for smoking status (never, former, or current), alcohol consumption (never, former, moderate, heavy), leisure-time physical activity (MET-hours/week), sedentary behavior (television hours/day), body mass index (kg/m^2^), energy intake (kcal/day), and number of chronic diseases (diabetes, cardiovascular disease, chronic lung disease, musculoskeletal disease, cancer, and depression). ^d^ Model 3: As Model 2 and additionally adjusted for fiber, carbohydrate, unsaturated fat, and sodium intake. ^e^ Model 4: Linear regression model adjusted for sex, age, and educational level (primary or less, secondary, or university). ^f^ Model 5: As Model 4 and additionally adjusted for smoking status (never, former, or current), alcohol consumption (never, former, moderate, heavy), leisure-time physical activity (MET-hours/week), sedentary behavior (television hours/day), body mass index (kg/m^2^), energy intake (kcal/day), and number of chronic diseases (diabetes, cardiovascular disease, chronic lung disease, musculoskeletal disease, cancer, and depression). ^g^ Model 6: As Model 5 and additionally adjusted for fiber, carbohydrate, unsaturated fat, and sodium intake.

**Table 3:** Associations of dietary vitamin D intake with changes in the components of the pain scale over 5 years in older adults.

	Vitamin D Intake ^a^			
	Tertile 1 (Lowest)	Tertile 2	Tertile 3 (Highest)	Per 1-µg/day Increment
**Pain severity**				
More severe pain vs. no change/less severe pain				
Cases/n	74/278	67/345	53/327	194/950
Model 1: OR (95% CI) ^b^	Ref.	0.68 (0.47,1.00) *	0.57 (0.38,0.85) **	0.92 (0.85,1.00) *
Model 2: OR (95% CI) ^c^	Ref.	0.69 (0.47,1.02)	0.55 (0.36,0.84) **	0.91 (0.84,0.99) *
Model 3: OR (95% CI) ^d^	Ref.	0.68 (0.46,1.00)	0.53 (0.35,0.81) **	0.91 (0.84,0.98) *
Change in pain severity				
n	278	345	327	950
Model 4: β (95% CI) ^e^	Ref.	−0.39 (−0.74,−0.04) *	−0.47 (−0.83,−0.11) *	−0.07 (−0.12,−0.01) *
Model 5: β (95% CI) ^f^	Ref.	−0.36 (−0.72,−0.01) *	−0.41 (−0.78,−0.04) *	−0.06 (−0.12,0.00)
Model 6: β (95% CI) ^g^	Ref.	−0.37 (−0.73,−0.02) *	−0.42 (−0.79,−0.05) *	−0.06 (−0.12,−0.00) *
**Pain frequency**				
More frequent pain vs. no change/less frequent pain				
Cases/n	103/278	100/345	85/327	288/950
Model 1: OR (95% CI) ^b^	Ref.	0.76 (0.53,1.07)	0.72 (0.50,1.03)	0.96 (0.90,1.02)
Model 2: OR (95% CI) ^c^	Ref.	0.75 (0.52,1.08)	0.72 (0.49,1.05)	0.96 (0.90,1.03)
Model 3: OR (95% CI) ^d^	Ref.	0.75 (0.52,1.08)	0.69 (0.47,1.03)	0.95 (0.89,1.02)
Change in pain frequency				
n	278	345	327	950
Model 4: β (95% CI) ^e^	Ref.	−0.41 (−0.75,−0.08) *	−0.50 (−0.84,−0.15) **	−0.07 (−0.12,−0.01) *
Model 5: β (95% CI) ^f^	Ref.	−0.39 (−0.73,−0.05) *	−0.47 (−0.82,−0.12) **	−0.06 (−0.12,−0.00) *
Model 6: β (95% CI) ^g^	Ref.	−0.40 (−0.73,−0.06) *	−0.45 (−0.81,−0.09) *	−0.06 (−0.12,−0.00) *
**Pain locations**				
More pain sites vs. no change/fewer pain sites				
Cases/n	73/278	72/345	54/327	199/950
Model 1: OR (95% CI) ^b^	Ref.	0.79 (0.54,1.15)	0.63 (0.42,0.94) *	0.94 (0.87,1.01)
Model 2: OR (95% CI) ^c^	Ref.	0.78 (0.53,1.15)	0.60 (0.40,0.92) *	0.93 (0.86,1.00)
Model 3: OR (95% CI) ^d^	Ref.	0.78 (0.53,1.14)	0.60 (0.39,0.92) *	0.93 (0.86,1.01)
Change in the number of pain locations				
n	278	345	327	950
Model 4: β (95% CI) ^e^	Ref.	−0.39 (−0.67,−0.11) **	−0.37 (−0.66,−0.08) *	−0.04 (−0.09,0.00)
Model 5: β (95% CI) ^f^	Ref.	−0.39 (−0.68,−0.11) **	−0.39 (−0.68,−0.09) *	−0.05 (−0.09,0.00)
Model 6: β (95% CI) ^g^	Ref.	−0.41 (−0.69,−0.12) **	−0.41 (−0.71,−0.11) **	−0.05 (−0.10,−0.00) *

* *p* < 0.05. ** *p* < 0.01. OR: odds ratio. CI: confidence interval. ^a^ Vitamin D intake categories: Tertile 1, 0.01 to 1.85 µg/day; Tertile 2, 1.85 to 3.51 µg/day; Tertile 3, 3.52 to 24.46 µg/day. ^b^ Model 1: Logistic regression model adjusted for sex, age, and educational level (primary or less, secondary, or university). ^c^ Model 2: As Model 1 and additionally adjusted for smoking status (never, former, or current), alcohol consumption (never, former, moderate, heavy), leisure-time physical activity (MET-hours/week), sedentary behavior (television hours/day), body mass index (kg/m^2^), energy intake (kcal/day), and number of chronic diseases (diabetes, cardiovascular disease, chronic lung disease, musculoskeletal disease, cancer, and depression). ^d^ Model 3: As Model 2 and additionally adjusted for fiber, carbohydrate, unsaturated fat, and sodium intake. ^e^ Model 4: Linear regression model adjusted for sex, age, and educational level (primary or less, secondary, or university). ^f^ Model 5: As Model 4 and additionally adjusted for smoking status (never, former, or current), alcohol consumption (never, former, moderate, heavy), leisure-time physical activity (MET-hours/week), sedentary behavior (television hours/day), body mass index (kg/m^2^), energy intake (kcal/day), and number of chronic diseases (diabetes, cardiovascular disease, chronic lung disease, musculoskeletal disease, cancer, and depression). ^g^ Model 6: As Model 5 and additionally adjusted for fiber, carbohydrate, unsaturated fat, and sodium intake.

## Data Availability

The datasets used and/or analyzed during the current study are available from the corresponding authors on reasonable request.

## Supplementary Materials

The following supporting information can be downloaded at: https://www.mdpi.com/article/10.3390/nu14183776/s1, Figure S1: Participants’ flow chart; Figure S2: Interaction analyses. Associations of vitamin D intake with pain incidence and changes in the pain scale over 5 years in older adults; Table S1: Dietary sources of vitamin D intake (µg/day) in older adults; Table S2: Sensitivity analyses. Associations of vitamin D intake with pain incidence and changes in the pain scale over 5 years in older adults.
