# Supplementary material for: Dietary Vitamin D Intake, Pain Incidence, and Pain Changes in Older Adults: The Seniors-ENRICA-1 Cohort
Source: Nutrients. 2022 Sep 13;14(18):3776. doi: 10.3390/nu14183776 (PMC9502822; doi:10.3390/nu14183776)
Supplement: Supplementary file 1 [file nutrients-14-03776-s001.zip › nutrients-1906503-supplementary.pdf]

Table S1. Dietary sources of vitamin D intake (μg/day) in older adults.

	**Mean (standard deviation)**	**%**
Total food	3.24 (2.47)	100
Fish	2.24 (2.41)	56.3 (28.9)
Oily fish	2.04 (2.37)	51.3 (30.0)
White fish and seafood	0.20 (0.70)	4.99 (14.4)
Eggs	0.39 (0.28)	17.5 (15.5)
Meat	0.17 (0.13)	8.37 (9.86)
Red and processed meat	0.11 (0.13)	5.54 (8.22)
Poultry	0.06 (0.05)	2.78 (4.04)
Offal	0.00 (0.01)	0.05 (0.56)
Cereals	0.23 (0.45)	8.21 (14.5)
Pastries	0.09 (0.25)	3.60 (9.10)
Whole-grain foods	0.07 (0.32)	2.09 (8.26)
Breakfast cereals	0.04 (0.20)	1.74 (8.05)
Cookies	0.01 (0.07)	0.59 (2.99)
Pasta	0.00 (0.03)	0.14 (0.89)
Bread	0.00 (0.01)	0.03 (0.28)
Dairy	0.14 (0.14)	6.66 (8.40)
Whole	0.12 (0.14)	5.59 (7.55)
Low-fat	0.02 (0.02)	1.07 (3.58)
Fats and oils	0.05 (0.20)	1.83 (6.53)
Butter and margarine	0.05 (0.20)	1.83 (6.53)
Oils	0.00 (0.00)	0.00 (0.02)
Vegetables	0.02 (0.10)	0.74 (4.21)
Sauces and condiments	0.10 (0.01)	0.20 (0.82)
Chocolate and cocoa	0.00 (0.01)	0.12 (0.50)
Nonalcoholic beverages	0.00 (0.00)	0.03 (0.21)
Coffee and tea	0.00 (0.00)	0.02 (0.21)
Other beverages	0.00 (0.00)	0.00 (0.04)
Snacks	0.00 (0.00)	0.02 (0.25)
Other foods	0.00 (0.00)	0.00 (0.00)

Table S2. Sensitivity analyses. Associations of dietary vitamin D intake with pain incidence and changes in the pain scale over 5 years in older adults.

	Vitamin D intake[a]			
	**Tertile 1 (lowest)**	**Tertile 2**	**Tertile 3 (highest)**	**Per 1-µg/day increment**
**Pain incidence**				
Cases/n	48/156	46/188	31/180	125/524
Adjusted for calcium intake: OR (95% CI)[b]	Ref.	0.78 (0.47,1.32)	0.49 (0.27,0.88)*	0.88 (0.79,0.99)*
Adjusted for sleep duration: OR (95% CI)[b]	Ref.	0.77 (0.46,1.31)	0.48 (0.27,0.87)*	0.88 (0.78,0.99)*
Adjusted for sleep quality: OR (95% CI)[b]	Ref.	0.78 (0.46,1.31)	0.49 (0.27,0.88)*	0.88 (0.79,0.99)*
Adjusted for vitamin D supplement use: OR (95% CI)[b]	Ref.	0.79 (0.47,1.32)	0.49 (0.27,0.88)*	0.88 (0.79,0.99)*
Cases/n	45/150	45/180	31/177	121/507
Excluding vitamin D supplement users: OR (95% CI)[b]	Ref.	0.84 (0.49,1.43)	0.52 (0.29,0.95)*	0.89 (0.80,1.00)*
**Changes in the pain scale**				
Pain worsening vs no change/pain improvement				
Cases/n	69/278	66/345	49/327	184/950
Adjusted for calcium intake: OR (95% CI)[b]	Ref.	0.73 (0.49,1.08)	0.55 (0.36,0.85)**	0.88 (0.81,0.96)**
Adjusted for sleep duration: OR (95% CI)[b]	Ref.	0.72 (0.48,1.07)	0.54 (0.35,0.83)**	0.87 (0.80,0.96)**
Adjusted for sleep quality: OR (95% CI)[b]	Ref.	0.72 (0.49,1.07)	0.55 (0.35,0.85)**	0.88 (0.81,0.96)**
Adjusted for pain medications use: OR (95% CI)[b]	Ref.	0.72 (0.48,1.07)	0.54 (0.35,0.84)**	0.88 (0.81,0.96)**
Adjusted for vitamin D supplement use: OR (95% CI)[b]	Ref.	0.72 (0.49,1.07)	0.56 (0.36,0.86)**	0.88 (0.81,0.96)**
Cases/n	64/265	61/326	48/318	173/909
Excluding vitamin D supplement users: OR (95% CI)[b]	Ref.	0.73 (0.48,1.09)	0.58 (0.37,0.91)*	0.89 (0.81,0.97)**
Change in the pain scale				
n	278	345	327	950
Adjusted for calcium intake: β (95% CI)[c]	Ref.	-0.56 (-1.01, -0.11)*	-0.56 (-1.03, -0.09)*	-0.07 (-0.15, 0.00)
Adjusted for sleep duration: β (95% CI)[c]	Ref.	-0.58 (-1.02, -0.13)*	-0.58 (-1.05, -0.11)*	-0.07 (-0.15, 0.00)
Adjusted for sleep quality: β (95% CI)[c]	Ref.	-0.57 (-1.02, -0.12)*	-0.56 (-1.04, -0.09)*	-0.07 (-0.15, 0.00)
Adjusted for pain medications use: β (95% CI)[c]	Ref.	-0.62 (-1.04, -0.20)**	-0.58 (-1.03, -0.14)*	-0.06 (-0.13, 0.01)
Adjusted for vitamin D supplement use: β (95% CI)[c]	Ref.	-0.57 (-1.02, -0.12)*	-0.56 (-1.03, -0.09)*	-0.07 (-0.15, 0.00)
n	265	326	318	909
Excluding vitamin D supplement users: β (95% CI)[c]	Ref.	-0.52 (-0.98, -0.06)*	-0.50 (-0.98, -0.02)*	-0.07 (-0.15, 0.01)

*p<0.05. **p<0.01. OR: odds ratio. CI: confidence interval.

[a]Vitamin D intake categories: Tertile 1, 0.01 to 1.85 µg/day; Tertile 2, 1.85 to 3.51 µg/day; Tertile 3, 3.52 to 24.46 µg/day.

[b]Logistic regression model as Model 3 in Table 2 [adjusted for sex, age, and educational level (primary or less, secondary, or university), smoking status (never, former, or current), alcohol consumption (never, former, moderate, heavy), leisure-time physical activity (MET-hours/week), sedentary behavior (television hours/day), body mass index (kg/m^2), energy intake (kcal/day), and number of chronic diseases (diabetes, cardiovascular disease, chronic lung disease, musculoskeletal disease, cancer, and depression), fiber, carbohydrate, unsaturated fat, and sodium intake].

[c]Linear regression model as Model 6 in Table 2 [adjusted for sex, age, and educational level (primary or less, secondary, or university), smoking status (never, former, or current), alcohol consumption (never, former, moderate, heavy), leisure-time physical activity (MET-hours/week), sedentary behavior (television hours/day), body mass index (kg/m^2), energy intake (kcal/day), and number of chronic diseases (diabetes, cardiovascular disease, chronic lung disease, musculoskeletal disease, cancer, and depression), fiber, carbohydrate, unsaturated fat, and sodium intake].

Figure S1. Participants' flow chart.

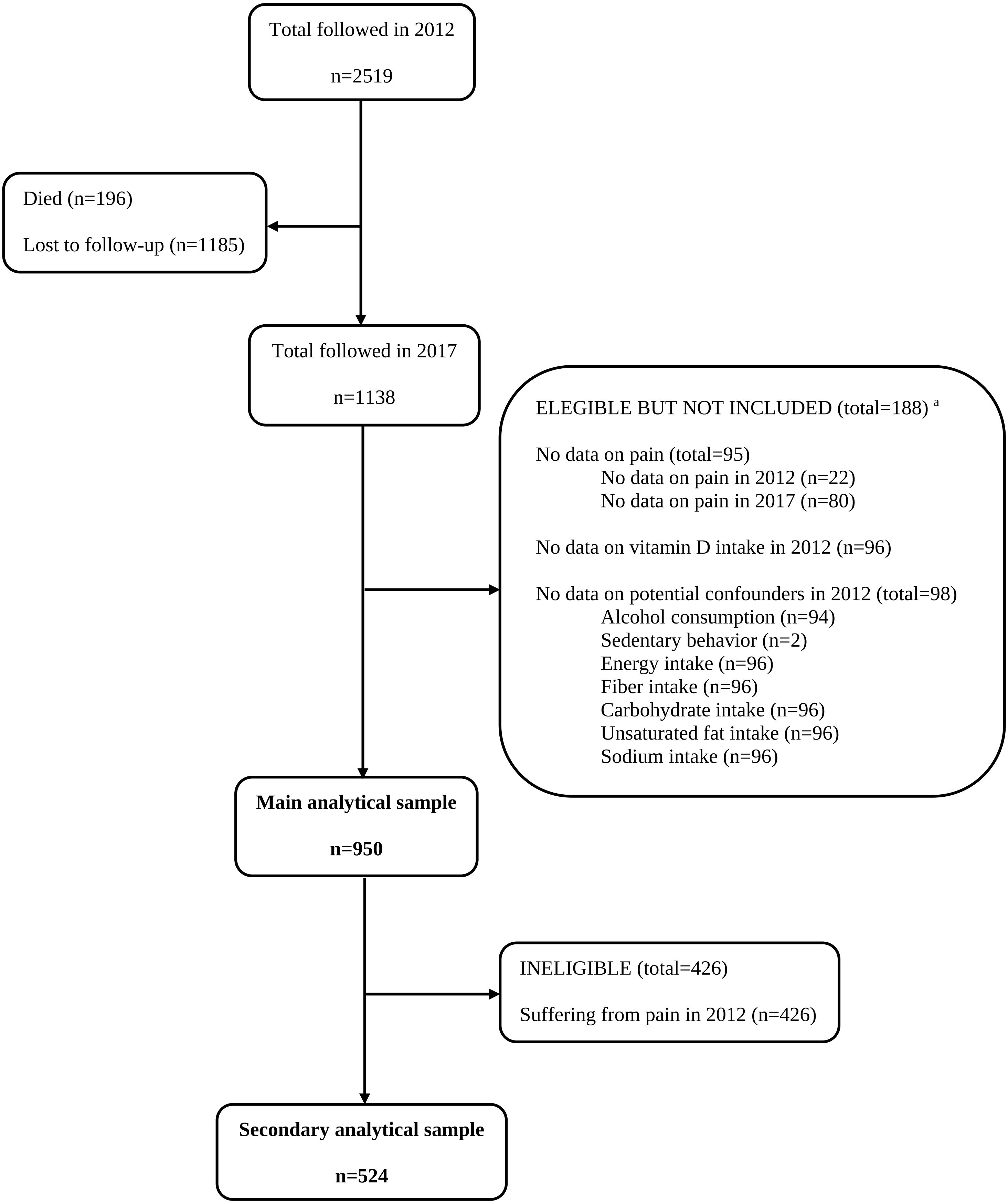

[a] Note that individuals may lack data in more than one variable.

Figure S2. Interaction analyses. Associations of dietary vitamin D intake with pain incidence and changes in the pain scale over 5 years in older adults.

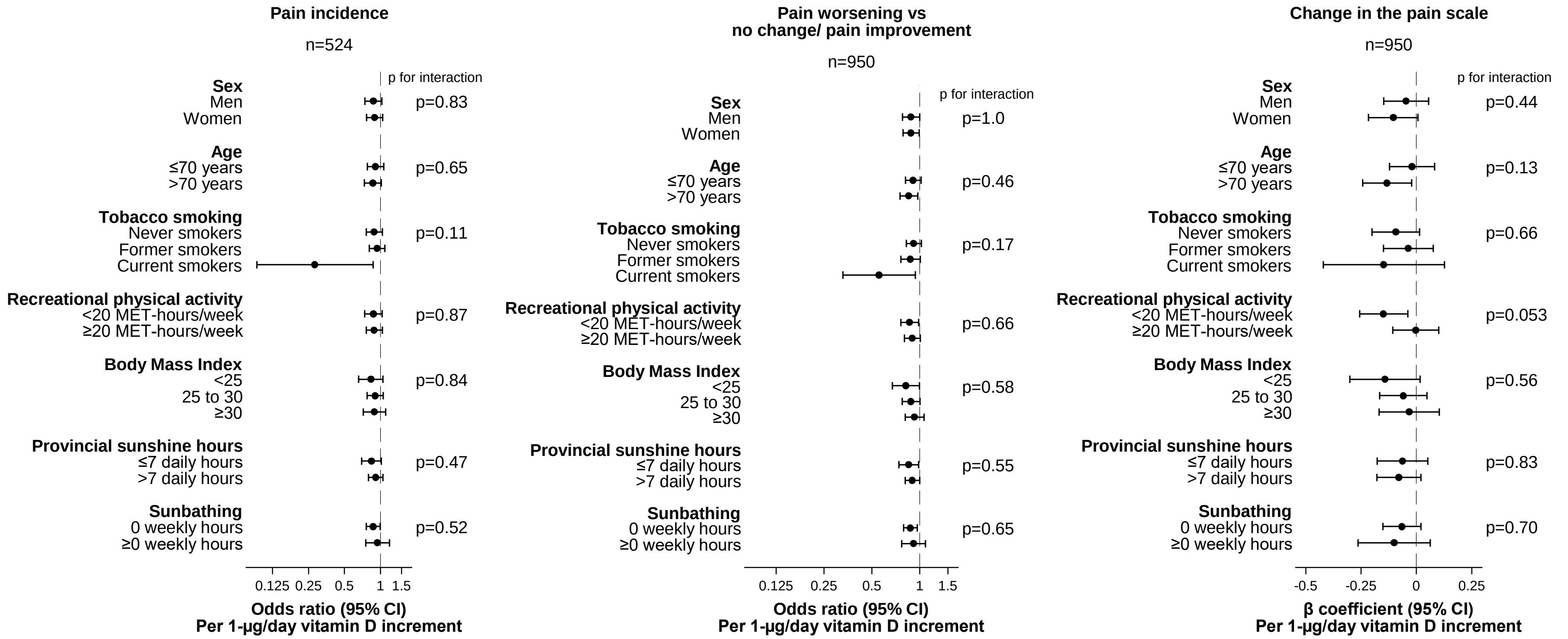

Plotted values are odds ratios (95% confidence intervals) from a model with interaction terms adjusted as the logistic regression Model 3 in Table 3, or β coefficients (95% confidence interval) from a model with interaction terms adjusted as the linear regression Model 6 in Table 3 [sex, age, educational level (primary or less, secondary, or university), smoking status (never, former, or current), alcohol consumption (never, former, moderate, heavy), leisure-time physical activity (MET-hours/week), sedentary behavior (television hours/day), body mass index (kg/m^2), energy intake (kcal/day), number of chronic diseases (diabetes, cardiovascular disease, chronic lung disease, musculoskeletal disease, cancer, and depression), fiber, carbohydrate, unsaturated fat, and sodium intake].

Wald tests that compared models with and without interaction terms were used to evaluate the statistical significance of such interactions.
